# Regional Barriers to Advancing Genetic Medicine in Japan: Insights from a Shizuoka Prefecture Survey

**DOI:** 10.31662/jmaj.2025-0240

**Published:** 2026-02-20

**Authors:** Kou Sueoka, Yuki Mizuguchi, Yasue Horiuchi, Takeshi Usui, Osamu Mochizuki, Hidehiko Miyake, Yuichi Goto

**Affiliations:** 1 Graduate School of Public Health, Shizuoka Graduate University of Public Health, Shizuoka City, Shizuoka Prefecture, Japan; 2 Department of Obstetrics and Gynecology, Keio University School of Medicine, Shinjuku-Ku, Tokyo, Japan; 3 Inagaki Ladies Clinic, Numadu City, Shizuoka Prefecture, Japan; 4 Ochanomizu University, Bunkyo-Ku, Tokyo, Japan; 5 National Center of Neurology and Psychiatry, Kodaira City, Tokyo, Japan

**Keywords:** designated intractable diseases, Board of Medical Genetics and Genomics, genetic counselor, online counseling, digital transformation

## Abstract

**Introduction:**

The expansion of genetic medicine in Japan has created an urgent need for regionally-adaptable systems to ensure equitable implementation. This study examines challenges in developing a sustainable framework for regional genetic medicine, using Shizuoka Prefecture―a non-metropolitan area with average socioeconomic indicators―as a case model.

**Methods:**

A two-phase survey was conducted between December 2023 and January 2024. The first phase involved a questionnaire sent to 44 major medical institutions and all board-certified clinical genetics specialists in the prefecture. In the second phase, 20 of the responding institutions completed a more detailed follow-up survey. The surveys assessed human resource capacity, institutional collaboration, and the adoption of digital infrastructure.

**Results:**

The results revealed critical shortages in clinical geneticists, certified genetic counselors, and genetic nursing specialists. Inter-institutional collaboration was limited, with few systems in place for data sharing or regional coordination. Digital tools, such as remote consultation systems and information platforms, were underutilized. Respondents identified the need for shared infrastructure, better communication among institutions, and flexible strategies to address geographic and systemic barriers.

**Conclusions:**

This study highlights the urgent need for network-based infrastructure and a specialized workforce to support the expansion of genetic medicine in regional settings. The findings from Shizuoka are likely reflective of broader national challenges and underscore the importance of policy and system-level interventions to promote equitable access to genetic healthcare across Japan.

## Introduction

Advancements in genetic medicine have revolutionized the diagnosis and treatment of hereditary diseases, offering new avenues for personalized care and improved quality of life ^(1)^. In Japan, for promoting genetic medical care, the Genome Medical Law was enacted as part of the national policy to promote treatment tailored to patients based on genetic information in 2023 ^(2)^. Prior to this political action, the Rare Diseases Act was established to address the many genetic-based rare diseases, and 338 specific rare diseases have been designated under this law up to 2023 as “Designated Intractable Diseases” approved by the Ministry of Health, Labour, and Welfare (MHLW) ^(3), (4)^. This support project has been established and expanded over the years. It was founded for providing financial assistance to patients, promoting research into causes and treatments, collecting epidemiological data, and enhancing specialist networks and care systems. The MHLW Research Project have been leading investigations with research institutions for diagnostics and treatment development efforts for each disease ^(5)^. Among the diseases targeted by genetic medicine, rare diseases often hold significant importance, including for the survival of patients. It is considered that there are approximately 9,000 types of rare diseases that affect fewer than 5 in 10,000 people, and 39% of rare diseases have known causative genes ^(6)^. As causative genes continue to be identified and reported at an increasing rate each year, the number is expected to increase further. As of 2022, 7,100 hereditary diseases had been registered, and with advancements in genetic analysis, the number continues to increase as previously unexplained diseases are further analyzed ^(7)^.

Despite these efforts, substantial regional disparities persist in the accessibility and quality of genetic medicine ^(8)^. A large proportion of designated diseases are managed at university hospitals and major urban institutions, limiting access in non-metropolitan areas. Moreover, the current disease registration system is based on primary symptoms rather than genetic diagnoses, hampering accurate epidemiological tracking and care planning ^(9), (10)^. Furthermore, identifying family members, including genetic carriers who face the risk of their future generations, presents even greater challenges ^(11), (12)^.

Shizuoka Prefecture, located centrally in Japan with average socioeconomic indicators and both urban and rural areas, presents a useful model for understanding regional challenges as a representative region of Japan. This study analyzes the current status of genetic medicine infrastructure in Shizuoka, identifying critical issues including specialist workforce, coordination among institutions, and usage of digital technologies. The goal is to extract lessons applicable to nationwide implementation strategies.

This study aims to identify structural, institutional, and personnel-related barriers to the promotion of genetic medicine in Shizuoka Prefecture, with the broader objective of informing national health policy. Specific goals are to clarify assessing the distribution of genetic medical services and specialists; evaluating the readiness of institutions to support genetic diagnosis and counseling; identifying gaps in inter-institutional cooperation and public information sharing, and proposing actionable recommendations for national and regional policy. This study primarily focused on rare diseases, while the survey was conducted without limiting the categories of conditions, including diseases such as cancer, which is currently introduced under national health insurance coverage.

## Materials and Methods

**Figure 1.**
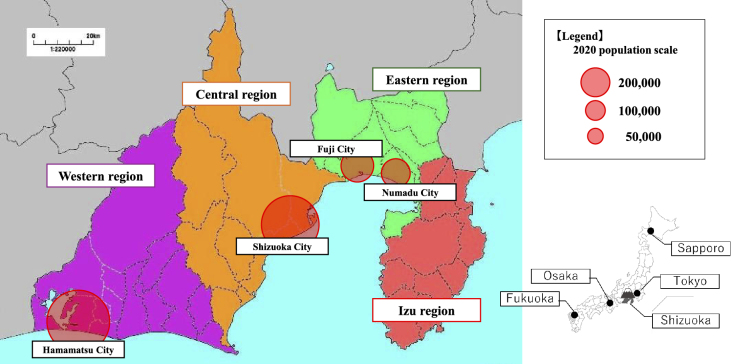
The geographical map of the areas in Shizuoka Prefecture and the cities. The survey was conducted in four areas consists of eastern, central, western, and Izu peninsula in the Shizuoka Prefecture. The cities and their population are depicted.

The survey was conducted from December 2023 until March 2024. An initial online questionnaire was sent to 44 major medical institutions futured in the list of hospitals in Shizuoka Prefecture and all medical facilities in which one or more registered certified clinical geneticists belong. A secondary detailed survey was conducted on 20 institutions that responded to the initial survey. Locations of the 44 surveyed medical institutions in Shizuoka Prefecture included 11 in the western region, 18 in the central region, 10 in the eastern region, and five in the Izu Peninsula region (Figure 1).

The questionnaire survey was conducted by first requesting participation via postal mail, followed by asking respondents to answer an online survey. The questionnaires were designed using multiple-choice and some descriptive formats to cover facility information, patient background, genetic medical systems, and medical collaboration.

The results from the first questionnaires were thoroughly examined and based on these results, the secondary survey was conducted to question in more depth, focusing on disease types, the capability of medical institutions to handle cases, referral situations to other facilities, the status of genetic testing, genetic counseling availability, geopolitical problems, and medical costs.

・Number of genetic medicine professionals at the facility and number of full-time positions (clinical geneticists, certified genetic counselors, certified genetic nurses).

・Availability and commitment to securing specialists.

・Clinical departments with physicians qualified as clinical geneticists.

・Establishment of departments or divisions specializing in genetic medicine.

・Clinical departments handling genetic disorders.

・Types and number of cases of genetic disorders handled in genetic medicine.

・Number of designated intractable diseases handled.

・Clinical departments handling designated intractable genetic disorders.

・Proportion of designated intractable diseases among all genetic disorders handled.

・Scope of services (testing, diagnosis, treatment, collaboration with other facilities).

・Residential areas of outpatient visitors, especially those receiving genetic medical care.

・Referring medical institutions.

・Referring facilities for patients with genetic disorders and designated intractable diseases.

・Number and destination of referrals of patients with genetic disorders to other facilities.

・Status of foreign patients.

・Targets and number of cases for genetic counseling.

・Status and challenges of telemedicine implementation.

・Opinions on the use of telemedicine for genetic medicine.

・Clients’ knowledge and understanding of genetics.

・Issues and challenges encountered in genetic counseling.

・Genetic counseling for foreign patients and languages used.

・Collaboration with specialized medical institutions.

・Need and mechanisms for information sharing among medical institutions within the prefecture.

In this study, information provided by the surveyed medical institutions excluded personal information that will lead to identification of patients or clients related to the diseases included in the survey. Conducted under the ethical review of Shizuoka Graduate University of Public Health, information about the study has been disclosed to allow for opt-out.

This research was supported by the Shizuoka Prefecture Social Health Medicine Research Project.

## Results

### The initial survey

#### Facility participation and characteristics

Out of 44 invited medical institutions, 20 responded to the initial survey, and 19 agreed to participate in the study. Among these, 17 institutions actively provided genetic medical services. A total of 16 facilities (84.2%) had at least one board-certified clinical geneticist (Certified Board Doctor of Clinical Genetics) on staff, either full-time or part-time, while three facilities provided services without certified specialists.

#### Regional distribution

Participating facilities were distributed geographically as follows: three in eastern Shizuoka Prefecture, nine in the central region, six in the western region, and one in the Izu Peninsula.

#### Departmental specialization and experience

Ten out of the 19 facilities (52.7%) had dedicated departments for genetic medicine. Of these, eight facilities (42.1%) reported over 10 years of experience in providing genetic services. Notably, three facilities offered genetic medicine without certified specialists.

#### Targeted diseases and services

**Figure 2.**
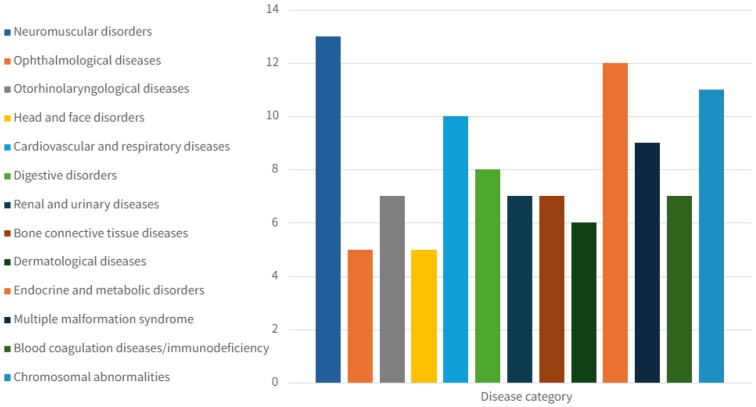
Types of genetic diseases treated at the medical facilities (multiple selections). This section presents aggregated data on the types of hereditary diseases managed by the surveyed medical institutions. The vertical axis indicates the number of institutions that have provided care for each disease category. The findings demonstrate that genetic services are being delivered for a wide range of disease types.

**Figure 3.**
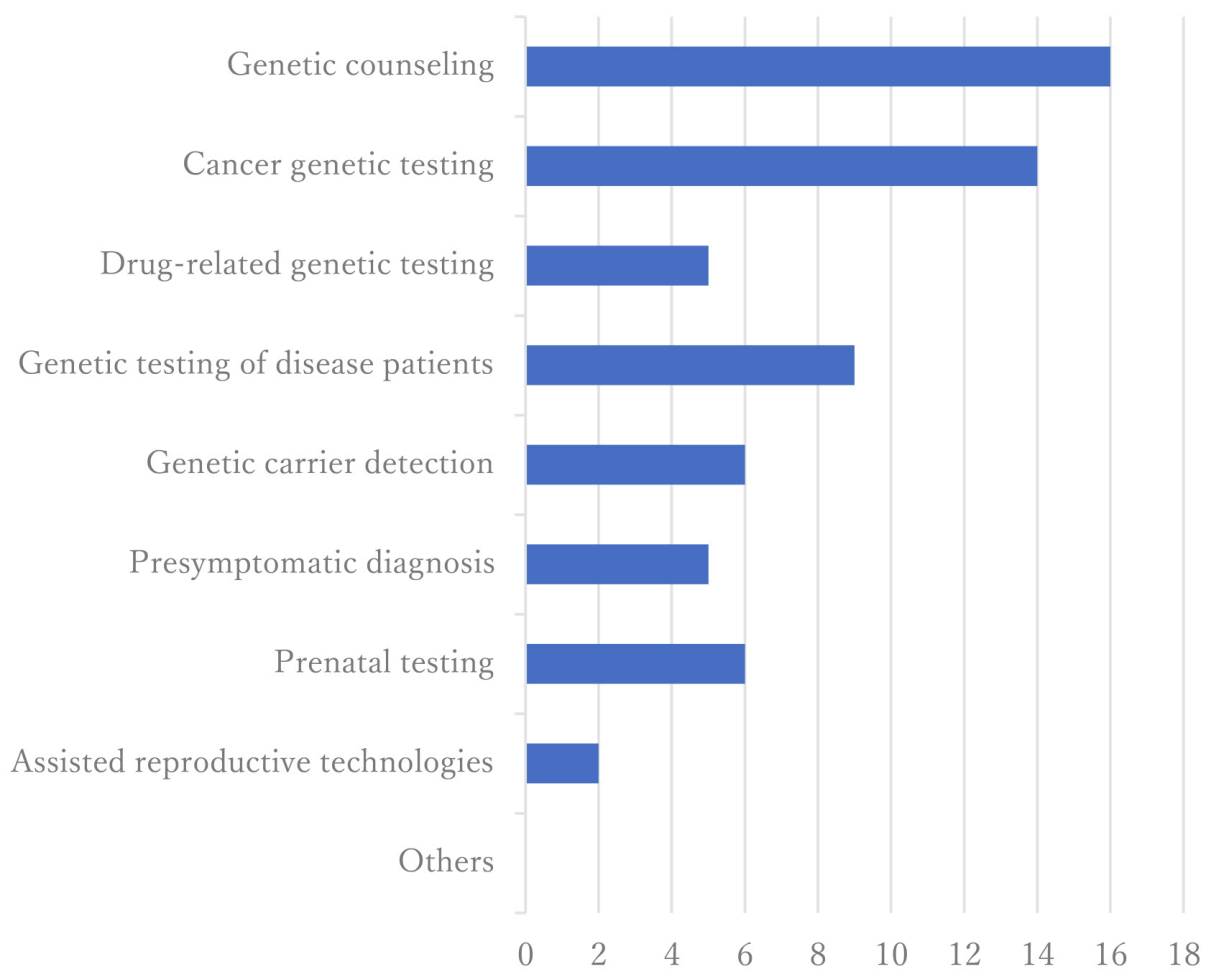
Types of genetic medical services provided at the facility (multiple selections). We also compiled the types of genetic services provided by each participating institution. The horizontal axis represents the number of institutions that have addressed each specific type of service.

Fourteen institutions (73.7%) managed hereditary diseases, while 12 (63.2%) handled designated intractable diseases recognized under Japan’s national healthcare support system. Common disease categories included neuromuscular, endocrine, chromosomal, and cardiovascular/respiratory disorders. Figure 2 illustrates the disease spectrum, and Figure 3 shows the types of genetic services offered.

#### Inter-institutional collaboration and service gaps

**Figure 4.**
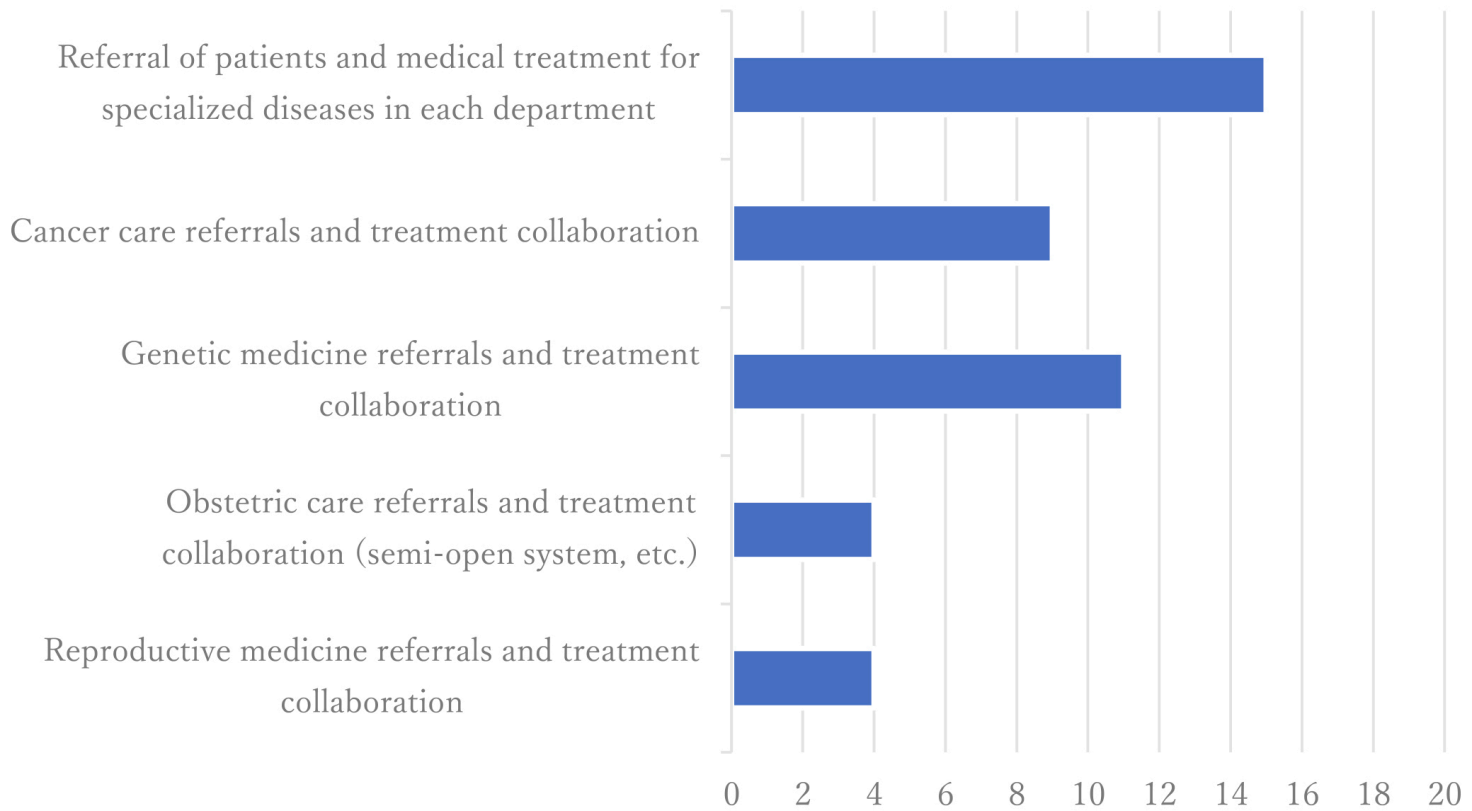
Items of collaboration with affiliated medical institutions (multiple selections). The identified services that individual institutions were unable to provide independently and instead referred to collaborating institutions. The chart shows the number of facilities that reported referring patients for each type of service, as represented on the horizontal axis.

Seventeen facilities (89.5%) reported collaborating with external medical institutions to compensate for in-house limitations, with university and general hospitals each comprising approximately one-third of collaborators. Nevertheless, 14 facilities (73.6%) had referred patients outside the prefecture due to the lack of local alternatives (Figure 4). Approximately half reported geographic barriers to service access, and only two institutions (10.5%) had implemented online genetic consultation services.

#### Key challenges identified

Primary concerns reported by 36.8% of institutions included the lack of publicly available information about other facilities, the absence of specialized centers, and insufficient nearby collaborative networks. Additional barriers cited included workforce shortages, limited financial incentives, and inadequate institutional recognition and support from administrators, often due to low awareness or engagement.

### Secondary survey

A follow-up, detailed survey comprising 116 items was conducted to obtain deeper insights into the current landscape of genetic medical services across Shizuoka Prefecture. Ten institutions responded. Their geographical distribution included six in the central region, three in the western region, one in the eastern region, and none in the Izu Peninsula.

All responding facilities had at least one Certified Board Doctor of Clinical Genetics, with staffing ranging from one to six per institution. In contrast, only four certified genetic counselors were reported, including part-time personnel. Notably, no facility employed a certified nurse specialist in genetics nursing.

**Figure 5.**
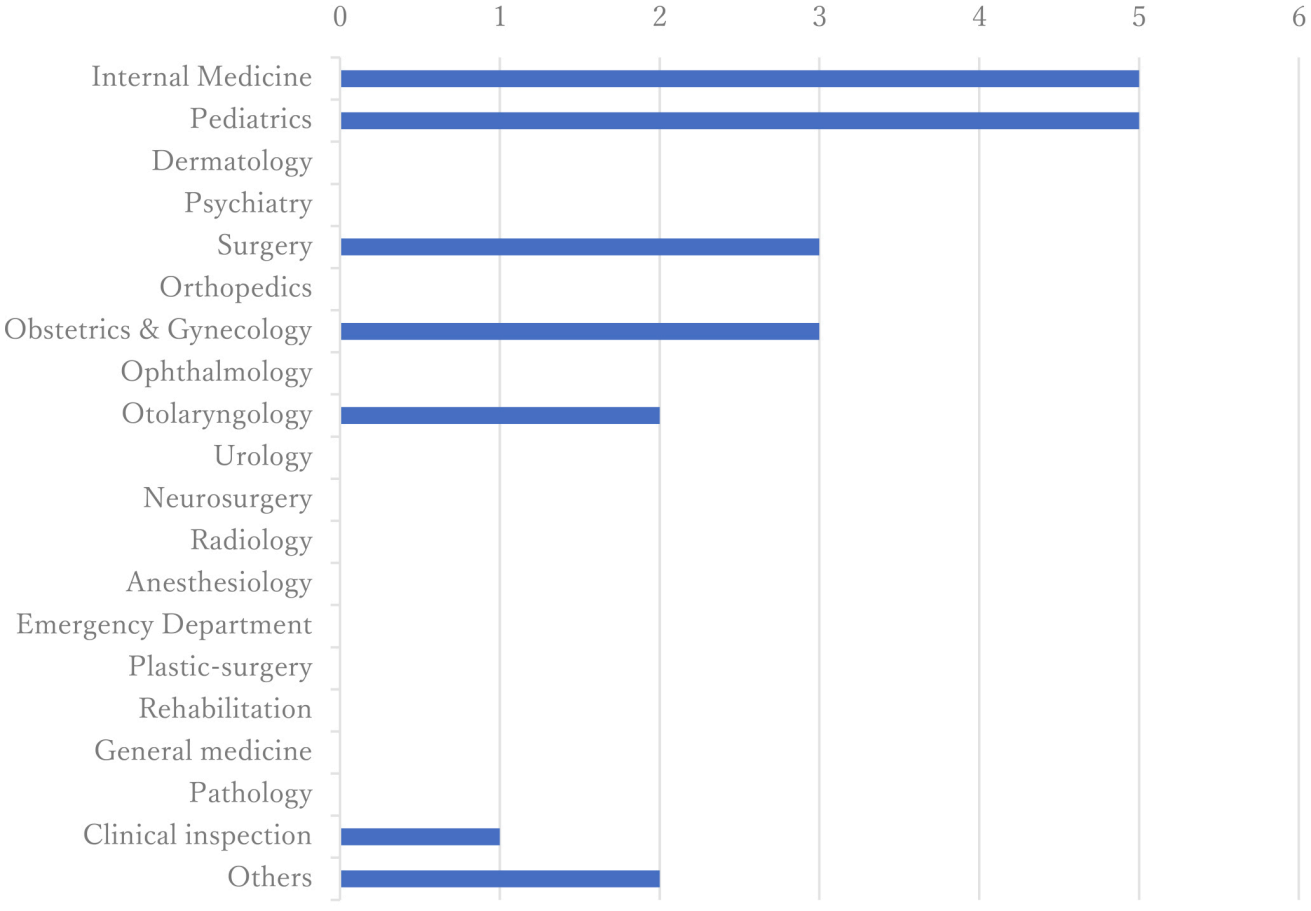
Department of Medical Practice provided support with board-certified doctors of clinical genetics. The number of institutions where clinical geneticists were involved in genetic consultations is also shown. The horizontal axis indicates the number of responding institutions. While many clinical departments are involved in genetic medicine, the departments where board-certified clinical geneticists were actively engaged remained limited.

**Figure 6.**
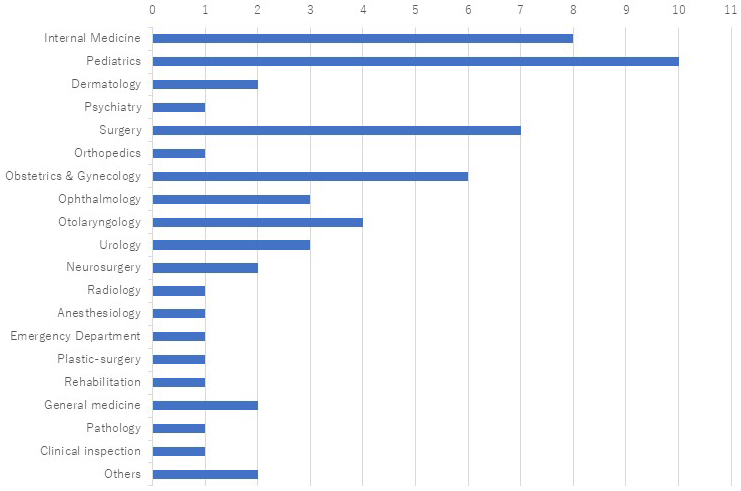
The fields of specialty departments dealing with genetic diseases. The horizontal axis represents the number of institutions offering care in each clinical field. While departments such as internal medicine, pediatrics, surgery, and obstetrics and gynecology were relatively well-represented, the results suggest that, despite the involvement of multiple specialties across a wide disease spectrum, certain conditions remain difficult to manage within the prefecture due to gaps in available expertise.

The areas of expertise and clinical responsibilities of genetic medicine professionals are presented in Figure 5, while the scope of medical fields involved is illustrated in Figure 6. Although these institutions represented a wide range of specialties, eight out of 10 reported limitations in initiating comprehensive genetic services. Annual case volumes varied widely, from as few as two to as many as 200 cases per facility. Nine of the 10 facilities managed designated intractable diseases.

All facilities reported referring certain complex cases to external institutions, including out-of-prefecture referrals. Cancer-related referrals were relatively limited (three facilities), and none of the institutions provided reproductive genetic services such as prenatal diagnosis or preimplantation genetic testing (PGT).

Regarding Digital transformation (DX), only one institution had implemented online consultations, despite some facilities citing geopolitical barriers. International patient interactions were reported, primarily involving clients from Brazil, the Philippines, China, and Vietnam. Counseling was generally conducted in Japanese or English, although interpreter assistance was required in some cases.

Eight of the 10 facilities reported difficulties in establishing efficient collaborative networks for managing diverse genetic conditions. Most required direct specialist-to-specialist contact across institutions. Seven facilities suggested that a shared information network across the prefecture would facilitate collaboration, while five expressed uncertainty and none opposed the idea.

If such a system were established, nine out of 10 institutions indicated they would actively use it. Proposed functions included streamlined patient referrals, access to other institutions’ service information, and integration of online consultation platforms.

**Figure 7.**
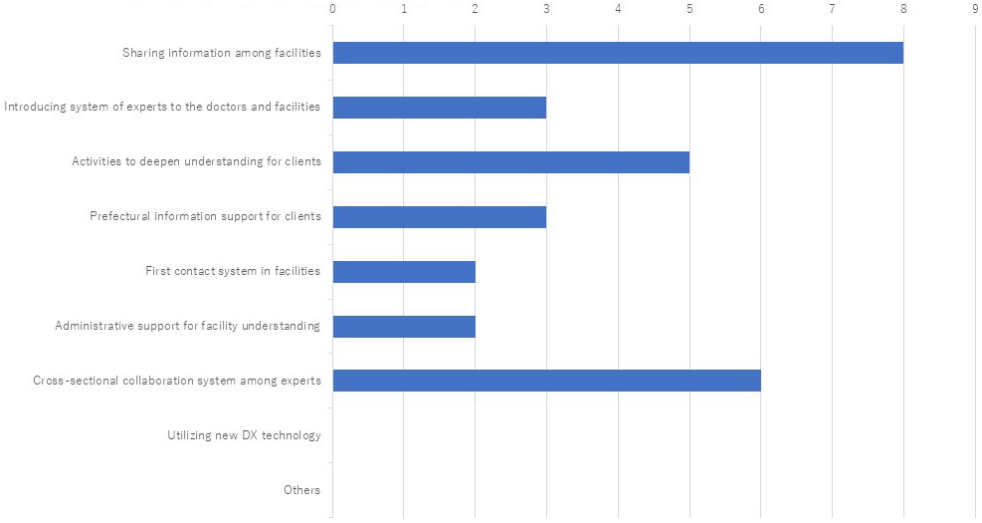
Opinions on specific measures for a system to deepen mutual understanding in response to the many opinions of lacking information between medical institutions in the prefecture. One of the major challenges identified in the provision of genetic medicine was the lack of information sharing between medical institutions. The results highlight the need to establish a coordinated system that facilitates inter-institutional collaboration, including patient referrals.

Responses from both the initial and secondary surveys highlighted a consistent lack of mutual visibility among institutions. Figure 7 summarizes key areas of demand, with the most frequently cited being: (1) knowledge about other institutions’ services, (2) improved understanding for patients and clients, (3) referral pathway transparency, and (4) better access to information provided by the prefectural government.

## Discussion

The initial survey of major medical institutions in Shizuoka Prefecture served as a representative overview of the regional status of genetic medicine. Facilities that did not respond or declined to participate were generally those without registered clinical geneticists, with only one exception. Conversely, the institutions included in the secondary survey were primarily those with a history of involvement in genetic medicine, providing a more detailed reflection of current capabilities and challenges within the field.

The results indicate that only a limited number of institutions are consistently engaged in specialized genetic medicine. Despite the broad spectrum of genetic disorders covered, a significant gap remains in the provision of care for certain rare diseases and advanced services such as reproductive genetics. This gap underscores the difficulty of distributing specialized expertise across 338 nationally designated intractable diseases in 2023 (341 in 2024) in Japan, particularly in fields involving inheritance and reproduction, such as prenatal diagnosis and PGT, which are related to inheritance to the next generation ^(11)^.

A critical issue identified is the shortage of specialized personnel, especially co-medical professionals, such as certified genetic counselors and nurses. This highlights the urgent need for human resource development as a regional priority. Combined with geopolitical challenges, such as uneven regional resource distribution, these factors hinder the formation of a sustainable medical care system and require targeted policy interventions.

Specialized expertise in genetic medicine, particularly in the context of rare diseases, is often unavailable in every region, making inter-institutional collaboration essential. In this regard, Shizuoka Prefecture offers a compelling case study. Located in central Japan, it is neither a highly resourced urban hub like Tokyo nor a medically underserved remote area, making it a useful model for identifying broader national issues.

In terms of demographics, Shizuoka Prefecture accounts for approximately 2.9% of Japan’s total population. In 2024, its total fertility rate (TFR) declined to 1.19, down from 1.25 in 2023, reaching a new historical low for the prefecture. Nationally, the TFR decreased to 1.15 in the same year, marking a record low for Japan. The prefecture is politically and demographically diverse, with distinct urban centers (e.g., Shizuoka City and Hamamatsu City) and rural areas facing ongoing depopulation and facility closures due to shortages in both population and healthcare personnel.

As of 2022, Shizuoka Prefecture had only 238.3 physicians per 100,000 population, which was well below the national average of 274.7 ^(13), (14)^. However, Shizuoka City and Hamamatsu City exceeded this average; 308.4 and 280.4 respectively, likely due to the presence of a medical school in Hamamatsu, which may be contributing to physician development locally. Nonetheless, many areas within the prefecture have historically relied on temporary staffing from major cities such as Tokyo, Nagoya, and Kyoto. Although improvements are ongoing, recruiting and retaining personnel with expertise in advanced genetic medicine remains a critical concern.

From a facilities standpoint, Shizuoka had 4.7 hospitals and 77.1 general clinics per 100,000 population, lower than the national averages of 6.5 and 84.2, respectively, in 2022 ^(15)^. Although these numbers indicate better performance than physician density, they still reflect an underlying shortage of specialized services.

As of 2024, Shizuoka had 60 Board of Medical Genetics and Genomics (clinical geneticists doctors), which was 3.17% of Japan’s national total, slightly above the prefecture’s population share (2.86%) ^(16)^. However, the situation for genetic counselors is more concerning: only four are officially registered in Shizuoka (1.03% of the national total of 388 in 2023), and even with additional part-time workers, the overall number remains critically low ^(17)^. Notably, no certified genetic nursing specialists are currently present in the prefecture (0 out of 24 nationally) ^(18)^.

This scarcity reflects a broader national issue. Compared with countries like the United States or the United Kingdom, Japan lags significantly in training human resources for genetic medicine, further intensifying regional disparities. Although these challenges are unlikely to be resolved in the short term, analyzing and addressing Shizuoka’s specific issues, especially in relation to geopolitical and demographic factors, may offer scalable solutions applicable to other regions in Japan.

One of the most prominent issues identified in this study was the lack of a shared, publicly accessible information network among medical institutions. This hinders the ability to respond effectively to patient and client needs prior to consultation. The DX tools, including telemedicine and centralized referral systems, have the potential to enhance coordination between institutions. However, adoption in Shizuoka remains minimal, only one facility reported implementing online consultation services, which was likely due in part to regulatory restrictions on telemedicine in Japan. The political act of Japan has delayed approval to introduce the online medical care up to the critical status of the coronavirus disease 2019 pandemic.

The importance of developing systems to support hereditary disease care is increasing ^(19)^. However, the structural rigidity of Japan’s medical system complicates innovation. Public healthcare offers universal coverage and affordability, but clinical practice gaps persist. For example, diagnosis and treatment for symptomatic patients are typically managed in public institutions under health insurance, whereas asymptomatic carriers and reproductive genetics services are often handled by private institutions, without or partly with insurance coverage. Most reproductive clinics are privately operated, and procedures such as PGT are paid entirely out-of-pocket by patients. Moreover, the legal prohibition on mixing insured and self-pay services further limits flexibility in patient care. Patients, accustomed to receiving covered services, often hesitate to pursue uninsured care, even when necessary.

This structure of national health insurance system is based on a commendable policy principle that ensures equitable access to healthcare for all citizens. It has significantly contributed to the achievement of a long-lived society. However, providing advanced medical care within the scope of public insurance poses considerable challenges in terms of healthcare economics. While the overall national healthcare budget is set annually by government authorities, securing financial resources has become increasingly difficult in recent years due to various factors, including population aging and a declining birthrate. As a result, there is growing pressure to contain rising medical costs. Although Japan’s total healthcare expenditure has remained relatively low compared to other countries, this dilemma has become a major subject of ongoing policy debate regarding the sustainability of the health insurance system.

These systemic constraints complicate the rapid integration of genetic services into routine practice. The financial burden of addressing each condition individually is substantial, and resource integration must be prioritized for sustainable growth ^(20), (21)^. Although swift progress in workforce development may be unrealistic, implementing shared networks to support information exchange and public access could provide immediate improvements. Such efforts would not only benefit patients, but also assist carriers and at-risk individuals who have lacked access to specialized consultation services and remain uncertain about their genetic risks.

In summary, this study identifies the urgent need for training genetic medicine professionals, establishing effective collaboration among institutions, and improving access to accurate information for both clients and healthcare providers. Shizuoka Prefecture serves as a viable model for understanding and addressing these issues on a national scale. A coordinated approach that combines human resource development with practical digital infrastructure is essential to advancing genetic medicine throughout Japan.

Advancing genetic medical care in Shizuoka Prefecture, and by extension, similar regions in Japan, requires urgent development of specialized human resources and the establishment of robust inter-institutional collaboration. The implementation of DX tools and a shared network infrastructure are particularly critical to overcoming current limitations. As a foundational step, systematic information sharing among medical institutions must be prioritized. Moreover, addressing regional disparities will require flexible, context-specific strategies that account for the unique geopolitical and demographic challenges faced by each area.

## Article Information

### Acknowledgments

We would like to express our heartfelt gratitude to the physicians at the medical institutions who cooperated with our research. We also extend our sincere thanks to the staff of the Shizuoka Prefecture Health Policy Division for their invaluable advice.

### Author Contributions

Kou Sueoka: Study design and overall coordination.

Yuki Mizuguchi: Data analysis.

Yasue Horiuchi: Analysis of the current status of genetic counseling practices.

Takeshi Usui: Analysis of regional specialized human resources.

Osamu Mochizuki: Collection of information related to reproductive medicine.

Hidehiko Miyake: Analysis based on input from certified genetics professionals across Japan.

Yuichi Goto: Analysis of data from the Ministry of Health, Labour and Welfare’s research on intractable diseases.

### Conflicts of Interest

None

### IRB Approval Code and Name of the Institution

With regard to project number UG23101, conducted under the Shizuoka Prefecture Commissioned Research Program in Health and Medicine, the study was reviewed by the Ethics Committee of the Graduate School of Public Health, Shizuoka. The committee determined that the study falls outside the scope of the *Ethical Guidelines for Life Science and Medical Research Involving Human Subjects*, and accordingly granted approval for its implementation.
